# Correction: Therapeutic itineraries and testimonies of COVID-19 patients in Manaus, the epicenter of the pandemic in the Brazilian Amazon

**DOI:** 10.1371/journal.pone.0340129

**Published:** 2026-01-02

**Authors:** Igor Castro Tavares, Denise Maria Guerreiro da Silva, Wagner Ferreira Monteiro, Kássia Janara Veras Lima, Darlisom Sousa Ferreira, Wuelton Marcelo Monteiro, Flávia Regina Souza Ramos

The Funding statement for this article is incorrect. The correct Funding statement is as follows: This study was funded by the Fundação de Amparo à Pesquisa do Estado do Amazonas (FAPEAM) under Call No. 038/2022 – PDPG/CAPES/FAPEAM – Coordinator/Financial Support. The funders had no role in study design, data collection and analysis, decision to publish, or preparation of the manuscript.
