# Supplementary material for: Therapeutic itineraries and testimonies of COVID-19 patients in Manaus, the epicenter of the pandemic in the Brazilian Amazon
Source: PLoS One. 2025 Jul 24;20(7):e0327127. doi: 10.1371/journal.pone.0327127 (PMC12289024; doi:10.1371/journal.pone.0327127)
Supplement: S1 File — This file contains additional verbatim quotes from study participants, organized according to the three main analytical themes presented in the Results section: 1. Initial responses to illness: family strategies, popular remedies, and shared uncertainty; 2. The paradox of the pandemic in the professional subsystem: fear, unpreparedness, and scientific denialism; and 3. Emotional experiences, spirituality, and perceptions of the COVID-19 vaccine. (DOCX) [file pone.0327127.s001.docx]

**Supplementary File – Additional Participant Quotes**

Manuscript ID: PONE-D-24-53418

Manuscript Title: *Therapeutic Itineraries and Testimonies of COVID-19 Patients in Manaus, the Epicenter of the Pandemic in the Brazilian Amazon*

**1) Initial responses to illness: family strategies, popular remedies, and shared uncertainty;**

*“We tried everything people mentioned… we took everything.” (P2)
“My husband, the one who stayed by my side all the time. And my children outside.” (P12)*

*“My son. I had COVID and live on the second floor and he carried me on his back... so the care was very fast and the sequelae were not great.” (P21)*

*“I stayed home. My saturation was around 77, 78... Then, I had a very light cough, a cough of those who are catching the flu.” (P5)*

*“All I felt was coughing, shortness of breath and the fever I got at night. Early on, I was sweating.” (P15)*

*“I had a lot of muscle pain (...). This medicine I took, hydroxychloroquine. Everything people talked about we took.” (P2)*

*“I have a bit of trouble remembering the medications. I bought everything they told me to buy.” (P13)*

*“First, I made a cocktail of medicines. I took dipyrone, dexamethasone and tylatil.” (P20)*

*“We tried everything to get better (...) mastruz... everyone brought everything and we took it.” (P2)*

*“The staff was making that jambu tea. I even drank it. When you are very bad, you believe and do everything, right?” (P5)*

**2) The paradox of the pandemic in the professional subsystem: fear, unpreparedness, and scientific denialism;**

*“I said, boy that's not a virus, no. Something's wrong. Then, when it was day two, she measured the saturation right? It was ninety-five, then she spoke like this - it's bad. We went to the emergency room (private service x). I have health insurance there ... so bad, no breathing right, no air. I took a car, I left for the public health service.” (P1)*

*“The symptoms I was feeling were similar to those of malaria, according to them, so I went home.” (P8)*

*“In the service at Z, they said that I didn’t have anything; why didn't they do a test for Coronavirus? They did a test for malaria because the symptoms I was feeling were similar to those of malaria.” (P28)*

*“When I arrived, in the emergency room they treated me a little rudely - oh, why don't I stay at home? There are a lot of people who are dying, it is full here.” (P17)*

*“The fever did not stop, aches in my body, headache, high blood pressure, taking medicine. I returned to the same clinic. I do not know how to tell you, because the person who was with me unfortunately later passed away.” (P4)*

*“They did the CT scan and found 70...80% saturation and I was hospitalized there, oxygen at high flow.” (P18)*

*“It was one hundred and sixty thousand [reais] to charter a plane, four days of waiting in line. We joined a line at the Office of the Public Defender. For patient transfer as well, but there wasn’t enough time.” (P2)*

*“I came to buy oxygen [...] There was a queue of cars, she went out because I no longer had oxygen. Asking permission, asking for God's sake. Then they let her pass.” (P16)*

*“When I arrived at the doctor’s office, the doctors were totally unprepared; all recent graduates [...] they had no medicine for blood pressure.” (P5)*

**3) Emotional experiences, spirituality, and perceptions of the COVID-19 vaccine.**

*“The feeling of helplessness, of death, your head goes through everything. Spend 25 days without seeing anyone, without looking at anyone, because you can't see [...] I think this ended up making people mentally ill.” (P5)*

*“I prayed a lot, and I asked God, not for me, but for my daughter because I have a teenage daughter.” (P3)*

*“You can't be a chaperone, right? So, I thought I'm going to die alone here.” (P1)
“Mentally, it ends up weakening the person. It ends up depressing them, weakening them more and more (...) The psychological aspect hits quite hard.” (P5)*

*“When you're a health professional, you know, you have some understanding about that. [... I'm evangelical, but I didn't let one thing interfere with the other. Because we know, right? There with our president and some pastors saying: - No, I will not vaccinate because it is (I am ashamed to say) it is the devil's work, the enemy, it is the mark [of the beast].” (P3)*

*“At first, even in the hospitals, we felt the health professionals themselves did not want to get vaccinated.” (P15)*
